# EZH2 in Bladder Cancer, a Promising Therapeutic Target

**DOI:** 10.3390/ijms161126000

**Published:** 2015-11-13

**Authors:** Mónica Martínez-Fernández, Carolina Rubio, Cristina Segovia, Fernando F. López-Calderón, Marta Dueñas, Jesús M. Paramio

**Affiliations:** 1 Molecular Oncology Unit, CIEMAT (Centro de Investigaciones Energéticas, Medioambientales y Tecnológicas), Avenida Complutense nº40. 28040 Madrid, Spain; carolina.rubio@externos.ciemat.es (C.R.); Cristina.Segovia@ciemat.es (C.S.); FernandoFeliciano.Lopez@externos.ciemat.es (F.F.L.-C.); marta.duenas@ciemat.es (M.D.); 2 Biomedical Research Institute I+12, University Hospital “12 de Octubre”, Av Córdoba s/n, 28041 Madrid, Spain

**Keywords:** bladder cancer, Polycomb, EZH2, miRNA, lncRNA

## Abstract

Bladder Cancer (BC) represents a current clinical and social challenge. The recent studies aimed to describe the genomic landscape of BC have underscored the relevance of epigenetic alterations in the pathogenesis of these tumors. Among the epigenetic alterations, histone modifications occupied a central role not only in cancer, but also in normal organism homeostasis and development. EZH2 (Enhancer of Zeste Homolog 2) belongs to the Polycomb repressive complex 2 as its catalytic subunit, which through the trimethylation of H3 (Histone 3) on K27 (Lysine 27), produces gene silencing. EZH2 is frequently overexpressed in multiple tumor types, including BC, and plays multiple roles besides the well-recognized histone mark generation. In this review, we summarize the present knowledge on the oncogenic roles of EZH2 and its potential use as a therapeutic target, with special emphasis on BC pathogenesis and management.

## 1. Introduction

Bladder cancer (BC) displays one of the highest incidences among different tumors in Western Europe. It also has a high prevalence due to the relatively low mortality. BC incidence is 3 times higher among men than women, being the 4th and the 11th most common cancer, respectively [1]. BC etiology is quite diverse. It is related with some factors like age, smoking, contact with some chemicals, or even with specific infections [2]. The infection of Schistosoma spp. is estimated to affect about 200 million people in the world. Although its mechanism of action remains unclear, it mostly produces squamous-like carcinomas [3]. It is estimated that >97% of all cases occur in endemic areas including Yemen, sub-Saharan Africa, Egypt and Sudan, but some cases have also been reported in Europe probably due to travels to these areas [4,5,6].

BC is classified depending on the stage (CIS (carcinoma *in situ*), Ta (Non-invasive papillary carcinoma), T1 (Tumor invades subepithelial connective tissue), T2 (Tumor invades muscularis propria), T3 (Tumor invades perivesical tissue) or T4 (tumor has spread beyond the fatty tissue and nearby organs or structures)), which reveals the location and invasiveness of the tumor. The grade (G1–3) evaluates the differentiation state of the tumor (from highly to poorly differentiated). This classification dates from 1973 and it is the most referenced worldwide [7]. However, there is a more recent classification, published by the World Health Organization (WHO) in 2004 [8]. This new classification (TNM) divides the tumors depending on the tumor-stage, on the invasion of the nearby lymph nodes, and on the existence of metastasis [9]. New classifications based on the expression patterns of different cancer-related proteins and genes have been recently proposed [10].

The Non-Muscle Invasive Bladder Cancer (NMIBC) (Ta-T1 stage) is the most frequent form of BC, representing up to 70% of newly diagnosed cases [11]. The other 30% represents Muscle Invasive Bladder Cancer (MIBC), from T2 to T4. This characteristic is of extreme relevance as it may define the potential treatment. Patients bearing NMIBC are usually treated by transurethral resection of the bladder tumor (TURBT), which allows bladder preservation and, according specific pathological conditions (size, number of implants, *etc.*), it is followed by local instillation with chemotherapeutics (mitomycin c, epirubicin, doxorubicin or gemcitabine) [2,12] or Bacillus Calmette–Guérin (BCG). BCG acts as a potential immunomodulator producing a local infection and a consequent inflammatory process, leaded by macrophages, Natural Killer cells, T-cells infiltration, and pro-inflammatory interleukin secretion that contributes to tumor eradication and prevent recurrence [13,14]. These adjuvant treatments require several doses for a long period of time, causing an important morbidity. Despite TURBT and chemo/immunotherapy, near 60% of the patients develop recurrences and, 15% of them, tumor progression to muscle invasive tumors [15,16]. Due to this, BC patients require continuous follow up by cystoscopy, making NMIBC one of the most expensive cancers for the National Health systems of European countries. MIBC treatment implies, in most of the cases, radical cystectomy and conventional chemotherapy with cisplatin, commonly combined with methotrexate, vinblastine and doxorubicin (M-VAC) or carboplatin combined with gemcitabine. The criteria to choose the most suitable treatment are diverse, but it is taken into account the renal function, the heart disease risk, obesity, and other comorbidities [12]. However, metastasis and mortality rates are very high, and survival expectancies are below 10% by 5-year in metastatic BC patients [17,18]. Remarkably, and in spite of its socioeconomic relevance, no new therapies have been adopted for the treatment of invasive BC for almost 20 years, probably because of a lack of substantial research support in comparison with other tumor types.

Molecular portrait of NMIBC and MIBC has led to the consideration that these two types of tumors arise as a consequence of distinct molecular alterations. In general, NMIBC has been associated with alterations in oncogenes such as *PI3KCA* (Phosphatidylinositol-4,5-Bisphosphate 3-Kinase, Catalytic Subunit α) and *FGFR3* (Fibroblast growth factor receptor 3) [19,20,21], whereas MIBC is assumed to proceed through tumor suppressor loss of function, particularly affecting *TP53* (Tumor Protein P53) and *RB1* (Retinoblastoma 1) genes [22]. These observations have been partially confirmed through the use of next generation massive genomic analysis of tumors [23,24]. Although these studies have been primarily focused in MIBC samples producing some bias of the findings, the results have provided a new landscape of BC molecular pathology, allowing a novel classification of bladder tumors as intrinsic subtypes, similarly to the concept developed in breast cancer and applied to a number of human malignancies [25]. The proposed intrinsic subtypes by three independent groups [25,26,27] display high relevance, as they correlate genomic profiles with the clinical outcome, and may represent a new future approach for the management of BC patients. More recently, the detailed analyses in independent datasets have suggested the molecular convergence to a phenotype that discriminate two major subtypes of BC [28].

In addition, the genomic characterization of BC samples has allowed the identification of novel pathways. Besides the “usual suspects”, BC is also characterized by the frequent alterations in DNA repair and in chromatin remodeling genes [24]. These observations may provide new future therapeutic avenues for the BC treatment.

Regarding the chromatin-remodeler genes, the involvement of Polycomb Repression Complex (PRC) has gained relevance in the last years, due to its implication in multiple malignancies [29,30,31,32]. PRC occurs in two different flavors involved in histone modification: Polycomb Repressor Complex 1 and 2 (PRC1 and PRC2) [33]. PRC2 is mainly composed by four different proteins in mammals: EED (Embryonic Ectoderm Development), SUZ12 (Suppressor of Zeste 12 Homolog), EZH2 and RBBP7/4 (Retinoblastoma Binding Protein 7/4). It is implicated in several processes like stemness, maintenance of cell identity, and cell differentiation, [34]. EZH2 is the catalytic subunit of PRC2 and catalyzes the trimethylation of K27 of H3 (H3K27me3) [35]. This epigenetic mark primarily generates the repression of gene expression of affected regions in the genome. The overexpression of PRC2 proteins is a common characteristic of various human tumors, including BC, and it is involved in the development and progression of these tumors [36].

The Polycomb Repressor Complex 1 (PRC1) is primarily responsible of the monoubiquitynation of H2AK119 and of chromatin compaction, and recognizes the H3K27me3 mark left by the PRC2 complex. The core is always formed by the RING1A/B protein. It can bind BMI1 (B lymphoma Mo-MLV insertion region 1 homolog), MEL18 (Melanoma Nuclear Protein 18) or NSPC1 (Nervous System Polycomb-1), and associates with CBX (Chromobox homolog) and HPH (Human PolyHomeotic) proteins. It has also been reported that the complex (RING finger protein 1A/B-BMI1) RING1A/B-BMI1 can form other PRC1-like complexes, whose function remains unclear [37]. As in the case of PRC2, increased expression and activity of PRC1 components is also a common hallmark of multiple human cancers [38,39].

## 2. EZH2 Biological Function

**Figure 1:**
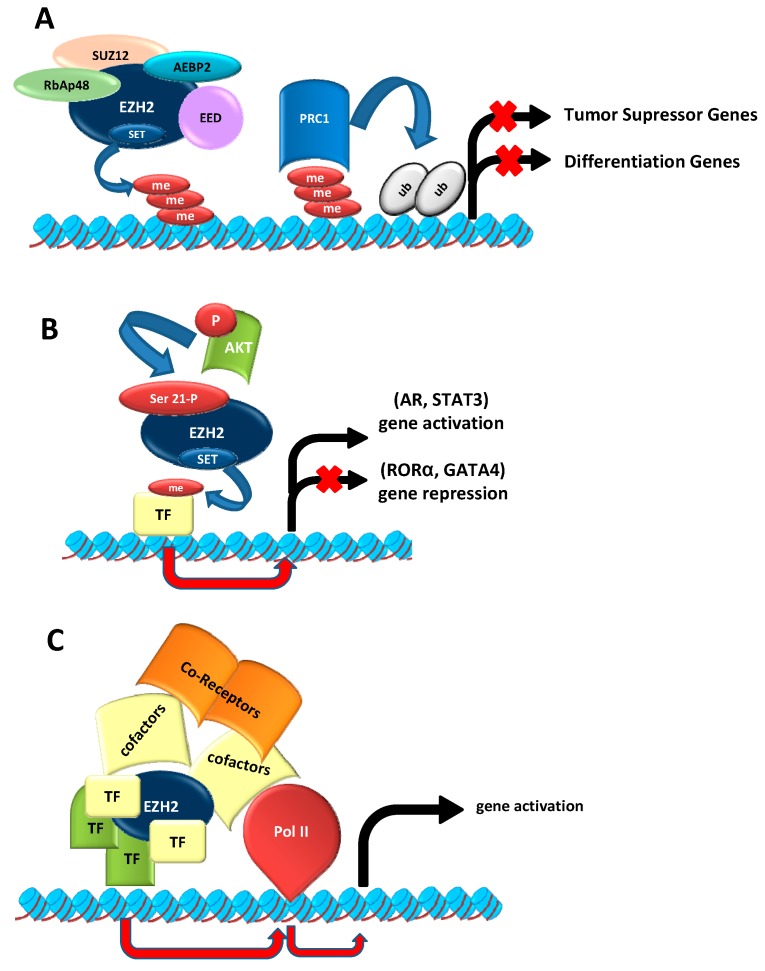
Canonical and non-canonical function of EZH2. (**A**) EZH2 as epigenetic silencer. EZH2 trimethylates lysine 27, and to a lesser extent, lysine 9 of histone H3 through its SET domain. These marks are recognized by PRC1, which monoubiquitynates lysine K119 on histone H2A, compacts the chromatin, and consequently represses gene transcription. In addition, EZH2 can induce tumor suppressor silencing and tumor progression; (**B**) PRC2-independent methylation of non-histone proteins. When EZH2 is phosphorylated by AKT in serine 21, it can methylate other proteins such as androgen receptor (AR), or transcription factors like Signal Transducer and Activator of Transcription 3 (STAT3), to activate gene transcription, or it can methylate other factors, such as Retinoid-related Orphan Receptor α (RORα) or cardiac factor GATA-binding factor 4 (GATA4), to repress transcription; and (**C**) Methyltransferase-independent EZH2 functions. EZH2 can act as scaffold protein for various transcriptional factors, such as estrogen receptors (ER) or components of the WNT/β-CATENIN signaling pathways to promote gene transcription (see references in the text). EZH2: Enhancer of zeste homolog 2; SUZ12: zing finger protein suppressor os zeste 12; EED: embryonic ectoderm development; SET: conserved domain Su (va) 3-9 Enhancer of Zeste and Trithoax; RbAp48: retinoblastoma-binding protein 48; AEBP2: adipocyte enhancer binding protein 2; AKT: is a serine-threonine specific protein kinase; TF: Transcription Factor. me: methylation; Ub: ubiquitynation; P: phosphorylation; Pol II: RNA polymerase II. The red arrows indicate induction of transcription. The black arrows mean the activation of gene transcription. The black arrow with a red cross means the repression of gene transcription. The light blue cylinders are histones, and the red ribbons are DNA.

The implication of EZH2 in tumorigenesis has been extensively documented in various types of tumors, including breast and prostate. Of note, its roles include not only epigenetic silencing through histone methylation, but also through gene expression activator of different pathways, and as a modulator of other cell proteins (Figure 1).

### 2.1. As Epigenetic Silencer

The EZH2 catalytic activity requires its SET domain [40]. Nonetheless, whole EZH2 activity needs EED and SUZ12, the two other subunits of the PRC2 complex, [41,42], which modulate the efficacy and the substratum preference, allowing the PRC2 allosteric regulation [43].

EZH2 may also cooperate with other epigenetic modifiers, such as DNA methyltransferases (DNMTs) [44] to promote a more permanent silencing of gene expression through CpG island methylation, and with histone deacetylases (HDACs) [45] allowing distinct histone methylation [33,46]. In this way, EZH2 acts coordinately with all these elements to silence genes involved in differentiation and cell cycle arrest , favoring stemness maintenance [47].

Interestingly, a novel EZH2 isoform, generated by alternative splicing and named EZH2β, has recently been characterized [48]. This isoform shares the gene silencing role with EZH2α, but it differs in its specificity for target genes, as EZH2α preferentially affects genes involved in cell cycle regulation and cell growth, while EZH2β regulates genes implicated in cell functions such as differentiation, angiogenesis, or stemness maintenance [48]. These differences in the specificity for target genes could be explained because both isoforms differ in a domain, absent in EZH2β, subject to various post-transcriptional modifications, including phosphorylation by different protein kinases. The existence of EZH2 isoforms provides a new panorama of the great complexity and plasticity of the remodeling chromatin mechanisms allowing a precise regulation of gene silencing [48].

The capacity of EZH2 to silence tumor suppressor genes or microRNAs justifies its consideration as an oncogenic factor in multiple cancers [49,50]. In this way, EZH2 can activate different oncogenic signaling pathways. For example, EZH2 can activate the non-canonical WNT signaling through the repression of DKK-1 (Dickkopf WNT signaling pathway inhibitor 1), an inhibitor of the WNT co-receptor LRP (Lipoprotein Receptor-related Protein) [51], may mediate the upregulation of RAF1-pERK-β-CATENIN pathway through RAD51 (DNA Repair Protein RAD51 Homolog 1) [52], inactivate BRCA1 (Breast Cancer gene 1) [53] or RUNX3 (Runt-related transcription factor 3) [54] expression. Finally, it can lead to RAS-ERK-AKT-NF-κB pathway activation through repression of tumor suppressor DAB2IP (Disabled Homolog 2-Interacting Protein) [55].

### 2.2. EZH2 Non-Canonical Roles

Interestingly, various studies have demonstrated that EZH2 not only acts as epigenetic silencer, but also it can act as a gene activator, independently on PRC2, through methylation of non-histone proteins favoring its oncogenic activity. For instance, in castration-resistant prostate cancer (CRPC), Xu *et al.* (2012) demonstrated that EZH2 phosphorylation in Serine 21 by AKT can switch its functions from transcriptional repression to gene activation [56]. This modification reduces its affinity for histone H3, allowing the androgen receptor (AR) methylation, or AR-associated proteins, triggering the transcription of specific subset of genes [57]. Similarly, this EZH2 phosphorylation allows the association with STAT3 promoting its methylation and activation in glioblastoma [58]. Remarkably, these novel actions may connect two oncogenic pathways, AKT and EZH2, which are supposed to act in an opposite manner regarding gene repression trough histone H3K27 trimethylation [57].

Other oncogenic activities associated with EZH2-mediated methylation of other non-histone proteins independently of PRC2 include the methylation-dependent nuclear receptor RORα degradation [59]. RORα has been considered a potential tumor suppressor, and its ubiquitin proteasome-mediated degradation may confer migratory and invasive properties to prostate cancer cells [60], facilitate WNT/β-CATENIN signaling in colon cancer [61], and activate proliferation in breast cancer cells [59]. Finally, EZH2 can methylate the transcription factor GATA (Globin Transcription Factor 4); this prevents its interaction with p300 histone acetyltransferase [62]. However, the possible relevance of this interaction in carcinogenesis remains unexplored and, in general, it is not yet known whether the processes of non-histone methylation are particularly restricted to prostate, breast or lymphoma tumors [63] or might be a more general characteristic that contributes to the overall oncogenic activities of EZH2.

EZH2 can also modulate gene expression independently of its histone methyltransferase activity. For instance in breast cancer cells, EZH2 can activate the transcription of different genes in two different manners, depending on the presence or absence of estrogen receptors (ERα). In ERα-positive luminal-like breast cancer cells, EZH2 can form transcription complexes with ERα or its co-activators, facilitating the TCF/β-CATENIN-mediated gene transcription [64]. On the other hand, in ERα-negative basal-like breast cancer cells, EZH2 can form complexes with RelA and RelB, activating the NF-κB pathway [65]. A similar process, favoring TCF/β-CATENIN-transcription, has been described in colon cancer cells, where EZH2 binds PAF transcription complexes [66]. Likewise, EZH2 can induce CYCLIN D1 expression in Natural Killer/T-cell lymphoma cells independently of its methyltransferase activity [67]. 

The importance of non-canonical function of EZH2 in BC still needs to be clarified. For instance, there are studies showing the STAT3 implication in MIBC [68,69,70], but its possible relation with EZH2 has not been demonstrated yet. In the case of WNT/β-CATENIN pathway, its activation through EZH2 in BC cells proliferation has been already described, but the exact activation mechanism is not known [71].

All these processes reinforce the role of EZH2 as an oncogenic factor, in particular associated with aggressiveness, progression and worse clinical outcome [47,72], as well as modulating cell plasticity and favoring intratumoral heterogeneity [73]. However, in spite that many of these signaling pathways are also involved in BC [68,69,70,71], the evidences of the involvement of EZH2 independently of its histone methyltransferase in BC are still scarce.

## 3. Regulation and Crosstalks

EZH2 expression can be regulated at multiple levels: it can be transcriptionally induced by multiple factors, such as by E2F family and C-MYC activation [74,75,76], or by the loss of p53 [77]; it can be also regulated post-transcriptionally through the interaction with microRNAs (miRNAs) and long non coding RNAs (lncRNAs) [78]. Moreover, its levels can be modulated through ubiquitination and degradation by the proteasome system, which are controlled by phosphorylation via PI3K-AKT [57,75] or CDK1 [79]. More recently, it has been demonstrated that EZH2 and SUZ12 can suffer sumoylation, although whether this may also affect their activity remains undetermined [80]. Remarkably, some of these mechanisms may act in various manners. For instance, C-MYC, besides inducing EZH2 transcription [76], can also modulate EZH2 through the induction of PTEN (Phosphatase And Tensin Homolog) expression, which in turn decreases the activity of AKT, thus reducing the inhibitory phosphorylation of EZH2 on Ser21 [57,75,81], and also represses the expression of various miRNAs that target EZH2 [82,83], or induces lncRNAs, such as HOTAIR (HOX transcript antisense RNA) [84], which functionally cooperate with EZH2 to repress gene transcription through H3K27me3 marks [85,86].

Regarding the regulation of EZH2 expression and/or activity by non-coding RNAs, special interest has been focused on miRNAs and lncRNAs. The miRNAs are small non-coding transcripts that participates in fundamental biological processes including development, apoptosis, differentiation, and proliferation [87,88]. Accordingly, miRNA expression appears deregulated in most, if not all, human cancers. MiRNAs also play important roles in post-transcriptional regulation of enzymes responsible of chromatin modification such as PRC, thus influencing chromatin structure [89,90].

The lncRNAs play a central role both in biological processes and pathological events [91,92,93]. These include nuclear trafficking (NORN) [94], genomic imprinting (Air, Kcnq1ot1) [95,96], and X-chromosome inactivation (Xist) [97]. Interestingly, both types of RNAs are also interconnected as lncRNAs can act as miRNA decoys, modifying their distribution on their targets [98]. Since lncRNAs have been proven to be in tight association with EZH2 (as we will discuss below in this review), and EZH2 regulates and is regulated by miRNAs, some authors have suggested that EZH2 may play a central role as a mediator in this lncRNA-miRNA-mRNA interaction [78].

### 3.1. MiRNAs-EZH2 Interactions

**Figure 2:**
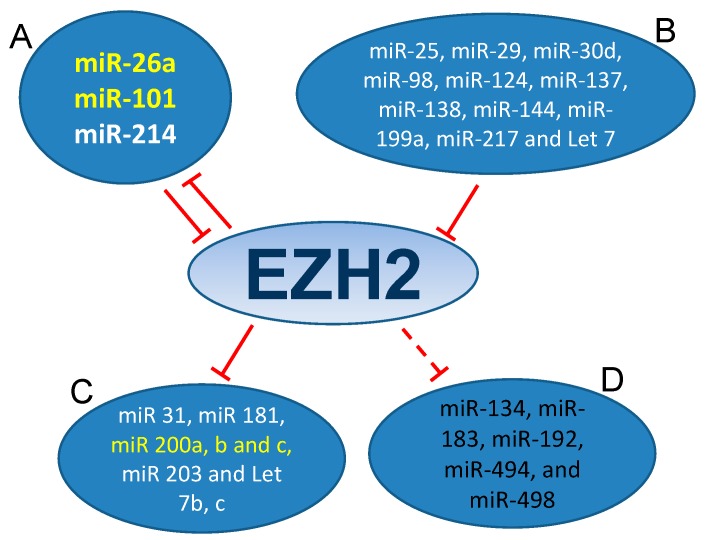
EZH2-miRNA network. The circles (**A**,**B**) show the miRNAs that regulate EZH2 by interacting with its 3′UTR. Circle (**A**) includes those miRNAs that, besides regulating EZH2, are also regulated/silenced by EZH2. The circles (**C**,**D**) indicate those miRNAs whose expressions are suppressed by EZH2 (**C**) or whose expression are silenced by hypermethylation (**D**). In white, the miRNAs with experimental evidence of EZH2 interaction; those that are also observed in BC are denoted in yellow; and in black those miRNAs without direct evidence of expression repressed by EZH2.

Several miRNAs have been demonstrated to be involved in EZH2 regulation. In Figure 2, we show a schematic representation of those miRNAs reported to interact with EZH2 in cancer development. Some of them directly regulated EZH2 post-transcriptionally, such as miR-101, miR-26a, miR-214, miR-217, miR-124, miR-138, miR-98, miR-25, miR-30d, miR-199a, miR-29, miR-144 and Let 7 family [71,83,99,100,101,102,103,104,105,106,107]. Among these, miR-101 has been found as negative regulator of EZH2 expression in BC [101,106,108,109,110]. The miR-101-EZH2 axis stems from previous work in mouse fibroblasts showing that during senescence EZH2 downregulation together with the histone demethylase KDM2B induces the expression of miR-101 [111]. In this system, the enforced expression of KDM2B caused the demethylation of H3K36 repressing the expression of miRNAs let-7b and miR-101, which in turn increased the EZH2 expression, contributing to cell immortalization. In BC cells, a similar axis involving NDY1/KDM2B-miR-101-EZH2 has been identified [112].

EZH2 is not only regulated by, but it also regulates a wide variety of miRNAs through epigenetic repression. These miRNAs may act as tumor suppressors, modulating tumor growth, a cancer stem cell phenotype, and cancer cell invasiveness. This EZH2-mediated repression has been demonstrated for miR-31, miR-200a-b-c, miR-181, miR-203, and Let 7b-c [50,111,113,114]. Among them, miR-181a-b, miR-200a-b-c and miR-203 are regulated by EZH2 and inhibit the expression of BMI1 and RING2, both PRC1 members. These findings suggest a possible regulatory axis including miRNAs-EZH2-miRNAs-PRC1 in advanced cancer [115]. The situation of miR-200 family members in this axis is of particular relevance in the context of BC. Liu *et al.* (2014) [116] reported that enforced expression of miR-200c in BC cell lines drastically reduced transcription factor E2F3, which acts as a positive activator of EZH2 and BMI1 transcription [74,117]. Interestingly, the two miR-200 clusters are concurrently silenced by promoter hypermethylation in advanced BC [118,119]. In addition, increased EZH2, which is a common hallmark of NMIBC at high risk of recurrence and tumor progression in recurrences [117], also caused a decrease of miR-200 family expression, and the knock down of EZH2 or its inhibition, using DZNep (3-Deazaneplanocin A), resulted in an increased expression of the miR-200 family in BC cell lines [108]. A similar effect of BMI1 suppressing miR-200 expression has been reported in other tumor types, such as breast and prostate [50] and in BC [119].

Of note, among the miRNAs that regulate EZH2, miR-26a, miR-101 and miR-214 have been proven to be repressed by EZH2. Varambally *et al.* (2008) [99] reported that the decreased expression of miR-101 correlated with high level of expression of EZH2 and H3K27me3 during prostate cancer progression. Last year, Wang *et al.* (2014) [82] described a feedback loop connecting EZH2, C-MYC and miR-101 leading to miRNA silencing in hepatocarcinogenesis. The molecular mechanism of this circuitry has been unveiled in B cell lymphomas [120]. In this system, EZH2 is recruited by C-MYC, epigenetically repressing the expression of miR-101. This downregulation of miR-101 expression increased expression of EZH2 and EED [120]. Similar circuitry has been demonstrated for miR-26a in Burkitt lymphoma cell lines supporting also the existence of an axis of MYC-miR-26a-EZH2-target genes in those lymphomas associated with MYC activation [83]. In the case of the miR-214 the regulatory feedback loop is direct. Juan *et al.* (2009) [100] reported that during skeletal muscle differentiation the miR-214 locus is activated by disengagement of PRC2 resulting in its transcription and when transcribed, miR-214 binds the 3′UTR region of EZH2 decreasing the EZH2 protein level.

Some miRNAs frequently associated to EZH2 regulation in several tumor types, such as bladder and gastric cancer, hepatocellular carcinoma and neuronal differentiation, include miR-214, miR-124 and miR-137 [120,121,122,123]. However, although these miRNAs are frequently downregulated in BC associated with increased stage and grade, no direct association has been conclusively reported between these miRNAs and EZH2 in bladder carcinogenesis [124,125]. Nonetheless, as EZH2 can also modulate DNA methylation (see below), it would be interesting to monitor the existence of direct correlation between EZH2 expression and the expression of these and other miRNAs reported to be epigenetically silenced by methylation in BC, such as miR-498, miR-494, miR-192, miR-183, and miR-134 [126].

### 3.2. LncRNAs-EZH2 Interactions

Long non-coding RNAs (lncRNAs) functions include to induce local gene silencing through binding to chromatin modification complexes, to recruit chromatin modifiers, and to act as scaffolds for chromatin modifying factors changing histone marks thereby modifying gene expression [78,127]. Their right functioning is fundamental for normal tissue maintenance, being their aberrant expression described in different human cancers [93,128,129].

Several lncRNAs have been shown to interact with PRC2, facilitating its recruitment to the promoter of some target genes. For instance, the *Prostate Cancer Associated ncRNA Transcript-1* (*PCAT-1*) is markedly increased in high-grade and metastatic prostate cancer. Its demonstrated interplay with PRC2 suggests that it could have an important role in prostate cancer progression [115]. 

Another example is the lncRNA *H19*. Luo *et al.* (2013) [130] demonstrated that lncRNA *H19*, already described as an enhancer of tumorigenic potential of carcinoma cells *in vivo,* promoted BC metastasis inhibiting E-CADHERIN expression and by associating with EZH2 .

The lncRNA *Up-regulated in Bladder Cancer 1* (*linc-UBC1*) also can be physically associated with PRC2 complex and appeared frequently upregulated in BC. It promotes increased cell proliferation, migration, invasion, metastatic potential, and its increased expression correlates with poor clinical outcome [131].

The lncRNA *Antisense Non-coding RNA in the INK4 (INhibitors of CDK4) Locus* (*ANRIL*) has been located within the p15/CDKN2B (Cyclin-dependent kinase inhibitor 2B)-p16/CDKN2A (Cyclin-dependent kinase inhibitor 2A)-p14/ARF (ADP Ribosylation Factor) gene cluster. It interacts with *EZH2*, *SUZ12*, and the PRC1 subunit *CBX7* (ADP Ribosylation Factors) mediating the silencing of *p16INK4a*, *p15INK4b* and *p14ARF* tumor suppressors through the recruitment of PRC2 and PRC1 [132,133,134]. In prostate cancer compared to normal prostate, its expression is increased and correlated with increased *EZH2* occupancy levels near the *INK4a* and *ARF* genes promoters [132]. However no differences have been recently observed in both NMIBC and MIBC, indicating a poor involvement in the development of this tumor type [24,135].

The most documented example of *EZH2*-lncRNA functional interaction is the *Homeobox Antisense Intergenic RNA* (*HOTAIR*). It is transcribed in antisense direction from the *HOXC* (Homeobox C) gene cluster and binds to *EZH2* recruiting PRC2 complex to specific target genes genome-wide. Kaneko *et al.* (2010) [85] and Tsai *et al.* (2010) [86] demonstrated that EZH2 interacts with *HOTAIR* to regulate gene expression. *HOTAIR* is frequently up-regulated in different cancer tissues, in particular in aggressive forms, and associated with metastasis development and poor clinical outcome [136,137,138,139]. Similarly, *HOTAIR* expression has also a prognostic value for BC recurrence, progression, and patient survival [135,140]. However, the functional roles of *HOTAIR* in modulating the cancer epigenome have not been completely elucidated. For example, in *HOTAIR*-deficient mice, the absence of this lncRNA causes H3K4me3 gain, whereas the effect on H3K27me3 is less evident [105]. Since *HOTAIR* also interacts with the *LSD1* (Lysine-Specific Demethylase 1) H3K4-specific demethylase, these findings may hamper the potential relevance of the *HOTAIR-EZH2* interaction *in vivo*. Whether these effects observed during normal mouse development could also similarly affect carcinogenesis, or if there are species-specific differences attributable to the disparity between mouse and human *HOTAIR* [141], remains to be elucidated.

### 3.3. EZH2 Interaction with Other Epigenetic Enzymes

Although PRC2 functions predominantly repressing target genes via the H3K27me3 repressive mark, other epigenetic mechanisms seem to be also involved in this gene silencing mediated by EZH2. Indeed, PRC2 components interact with DNMTs (DNA methyltransferases) and HDACs [142]. The DNMTs expression (DNMT1, DNMT3A, and DNMT3B) leads to gene inactivation by catalyzing the methylation of cytosine at CpG sites in the regulatory sites of target genes. EZH2 can bind and recruit DNMT1, DNMT3A and DNMT3B to their targets [44]. Moreover, EZH2 is required for binding of DNMTs and facilitates CpG methylation of EZH2-target promoters [44]. This process adds complexity and plasticity to the epigenetic program in which methylation of histones and DNA cooperates in an integrating gene-silencing network within the cell. In the cancer context, this interaction has been less explored. Indeed, the possibility that to reprogram the cancer epigenome both mechanisms can act in parallel has been already postulated [143]. Accordingly, the polycomb repressive mark and DNA methylation can act separately in subset of genes, whereas in other genes there is an “epigenetic switching” from a plastic polycomb mark to the more stable silencing by DNA methylation [143]. As a consequence, this switch does not lead to *de novo* repression but might significantly cause a reduction in the epigenetic plasticity, leading to a permanent repression of key regulatory genes and contributing to tumor development and/or progression. 

In this regard, the concerted action of DNMT1 and EZH2 mediates the repression of the miR-200a,b/429 locus contributing to the progression of gastric cancer and glioblastoma [144]. Interestingly, we have also recently reported that these miRNAs display reduced expression during BC progression by promoter methylation and/or EZH2 expression [119], suggesting that this repression could be mediated by EZH2-DNMTs collaborative activity. This represents an attractive possibility for future analyses, as they might also provide new therapeutic strategies for BC management.

Besides the H3K27me3, other well-characterized histone methylation event is the H3K9me2/3, a hallmark of constitutive heterochromatin. This methylation is catalyzed by specific histone methyltransferases (HMTs), including G9a (EHMT2), GLP (EHMT1) and SUV39H (Suppressor of Variegation 3–9 Homolog). Interestingly, although H3K9me2/3 and H3K27me2/3 marks were first thought to have distinct functions and to be mutually exclusive in genome, recent studies show the co-occupancy of these repressive marks at several gene loci [145,146], and a cooperative mechanisms for both methylation marks in keeping gene silencing in part through the recruitment of other protein partners [147,148]. More interestingly, the physical interaction between PRC2 core components and G9a/GLP has been recently described [149] allowing G9a/GLP to control the PRC2 recruitment and H3K27me3-mediated silencing at specific genomic loci [149]. The deregulated expression of G9a/GLP complexes in many tumors including hepatocellular, colon, prostate, lung, bladder, and in B cell chronic lymphocytic leukemia [150] possess a new possible mechanism contributing to PRC2 roles in tumorigenesis. This hypothesis still remains to be confirmed.

On histone tails, lysine residues can also undergo acetylation, resulting in a more open chromatin configuration [151]. Histone acetyl transferases (HATs) and deacetylases (HDACs) catalyze the dynamic regulation of acetylation. They add or remove, respectively, acetyl groups from histone tails. The deregulated expression of the various HDACs has been described in different cancer types, including urothelial tumors [152]. However, the variations in the expression of these isotypes, being some of them upregulated whereas others are repressed, makes difficult the use of general inhibitors, such as vorinostat, in the management of BC [153,154]. PRC2 could recruit HDACs through EED leading to cooperative repression of gene expression [155,156]. In addition, HDACs could deacetylate H3K27, allowing the subsequent methylation by PRC2, or could also deacetylate other histone lysines to adjust the local histone code for silencing, in a process similar to that described for Nucleosome Remodeling Deacetylase (NuRD) [42].

On the other hand, lysine-specific demethylases (KDMs) counteract different histone methyltransferases including EZH2 [111,157]. Several human isoforms KDMs have been already described [158,159]. The JmjC (Jumonji C) domain-containing proteins, *UTX*/KDM6A (Lysine (K)-Specific Demethylase 6A) and *JMJD3*/KDM6B (Lysine (K)-Specific Demethylase 6B), can particularly remove di- and trimethyl marks from H3K27 and acting against the histone modification mediated by EZH2 [160,161,162,163]. KDM6A was the first described mutated histone demethylase gene in human cancer [164], and most mutations cause loss of function [165]. Importantly KDM6A is among the most frequently mutated genes in BC [166,167] and its frequent loss of function also reinforces the oncogenic roles of EZH2 in these BC tumors, as the EZH2 activity (as histone methyltransferase) is opposed by that of KDM6A. In contrast to KDM6A, and in spite of having similar enzymatic function, no significant mutations or changes in expression have been reported for *JMJD3*/KDM6B in BC. In this regard, opposite functions of KDM6B and KDM6A have been demonstrated in the T-cell acute lymphoblastic leukaemia (T-ALL) [168], being KDM6B essential to initiate and maintain these tumors, whereas KDM6A acts as a tumour suppressor, being frequently genetically inactivated. The possibility that similar opposite functions also happen in BC needs further detailed studies.

## 4. Pharmacological Treatment

**Figure 3:**
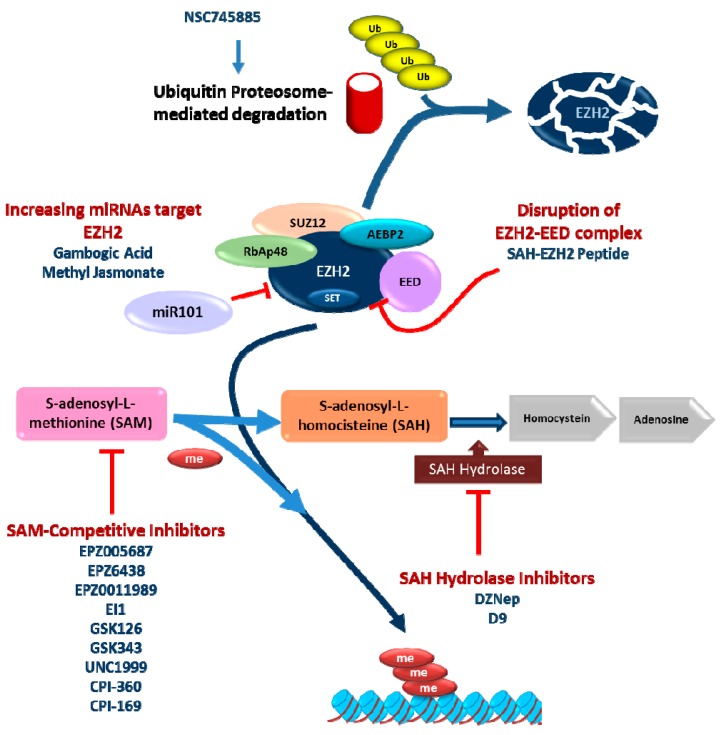
Inhibitors developed against EZH2. The five types of inhibitors are indicated: “*S*-adenosyl-l-homocysteine hydrolase inhibitors”, “*S*-adenosyl-l-methionine competitive inhibitors”, “*S*-adenosyl-l-homocysteine-EZH2 peptides as disruptors of the contact between EZH2 and EED”, “Degradation by ubiquitin proteasome pathway”, and “Downregulation mediated by the increased expression of miRNAs target of EZH2”. EZH2: Enhancer of zeste homolog 2. SUZ12: Zinc finger protein suppressor of zeste 12. EED:WD40 Repeat protein embryonic ectoderm development. SET: Su(var)3-9E Enhancer of zeste and Trithorax RbAp48: Retinoblastoma-binding protein p48. AEBP2: Adipocyte Enhancer-Binding Protein 2. DZNep: 3-dezaneplanocin-A Me: methylation. Ub: ubiquitination Mir101: miRNA-101. Arrow blue: activation of different pathways Arrow red: inhibition of different molecules. Light blue cylinders are histones, and with red ribbons are DNA.

As described above, EZH2 represents an oncogenic signal in several types of cancer, as prostate, liver, breast, colon, skin, lymphoma, endometrial, lung, myeloma, gastric, and BC [42,169,170]. Therefore, its inhibition may be a key approach for cancer treatment. This fact has inspired various pharmaceutical companies and academic research groups to develop inhibitors of EZH2 (Table 1 and Figure 3). Such inhibition of EZH2 could be achieved through direct inhibitors or through indirect mechanisms.

**Table 1:** Inhibitors developed against EZH2.

Name	Structure	Mechanism	Specificity to EZH2	Clinical Status	References
DZNEP	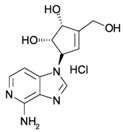	SAH Hydrolase Inhibitor	No	Preclinical	[171,172,173]
D9	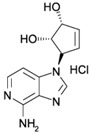	SAH Hydrolase Inhibitor	No	Preclinical	[174]
EPZ005687	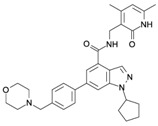	SAM-Competitive Inhibitor	Yes	Preclinical	[175]
EPZ-6438	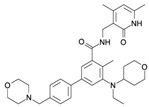	SAM-Competitive Inhibitor	Yes	Phase I/II trial	[176]
EPZ0011989	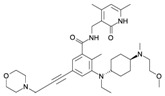	SAM-Competitive Inhibitor	Yes	Preclinical	[177]
EI1	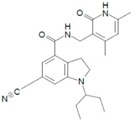	SAM-Competitive Inhibitor	Yes	Preclinical	[178]
GSK126	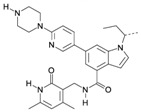	SAM-Competitive Inhibitor	Yes	Phase I trial	[179]
GSK343	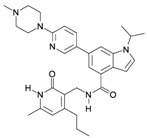	SAM-Competitive Inhibitor	Yes	Preclinical	[180,181]
UNC1999	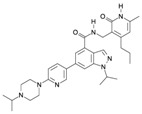	SAM-Competitive Inhibitor	Specificity to EZH1/2	Preclinical	[182,183]
CPI-360	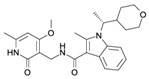	SAM-Competitive Inhibitor	Yes	Preclinical	[184]
CPI-169	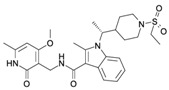	SAM-Competitive Inhibitor	Yes	Preclinical	[184]
SAH-EZH2 Peptide	Peptide: FSSNRXKILXRTQILNQEWKQRRIQPV	Disrupts the EZH2-EED complex	Yes	Preclinical	[185]
NSC745885	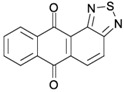	Degradation of EZH2 by proteasome	Yes	Preclinical	[186]
GA and MJ	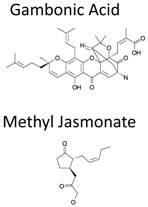	Down-regulation of EZH2 expression by mir-101 up-regulation	Yes	Preclinical	[187]

SAH: *S*-adenosyl-l-Homocysteine; SAM: *S*-adenosyl-l-methionine; SAH-EZH2: Stabilized-α-helix of EZH2.

### 4.1. S-Adenosyl-l-homocysteine Hydrolase (SAH Hydrolase) Inhibitors

The EZH2 protein contains different domains: H1 and H2 domains conform the binding region for PHF1 and SUZ12 respectively; cysteine rich domain, SANT domain to interact with histones; and C-terminal SET domain [63]. The catalytic activity of EZH2 resides in the SET domain [170]. It catalyzes the methyl group transfer from a universal methyl donor, *S*-adenosyl-l-methionine (SAM), to the lysine side chains of the acceptor protein. This generates S-adenosyl-l-homocysteine (SAH), which is further processed by the *S*-adenosyl-l-homocysteine hydrolase (SAH Hydrolase) [188].

One of the best characterized EZH2 inhibitors is 3-dezaneplanocin-A (DZNep), an equivalent of 3-deazaadenosine. It blocks the activity of SAH Hydrolase [170,189], producing the accumulation of SAH in cells and thus overall reduction of SAM, causing the indirect inhibition of the EZH2 methyltransferase activity. In addition, DZNep also causes the degradation of EZH2 probably through the ubiquitin-proteosome system [171,190]. These observations might support the widely reported preclinical antitumoral activities of this compound [172,173,191,192,193,194]. Although there is no data about how to predict its response in BC, it has been recently described TP53 genomic status influences DZNep response in gastric cancer [193]. However, current studies indicate that DZNep act as a global inhibitor of histone methylation, and probably inhibits diverse methyltransferases, in agreement with its pharmacologic impairment of SAH Hydrolase activity and the overall reduction of SAM [172]. Therefore, DZNep fails to be a specific and selective EZH2 antagonist [189,195].

This lack of specificity has led to the development of DZNep analogs with improved characteristics. One of them, named compound D9 has shown effectiveness in acute myeloid leukemia cells through the inhibition of various oncogenic signaling pathways [174]. Moreover, D9 can deplete the leukemia stem cells (LSC) and abolish chemotherapy-induced LSC enrichment, producing dramatic elimination of acute myeloid leukemia (AML) cell survival [174]. Therefore, in spite of the undetermined precise mechanism of action and the lack of *in vivo* data, D9 is becoming an interesting drug candidate thanks to its anti-cancer effects.

### 4.2. S-Adenosyl-l-methionine (SAM) Competitive Inhibitors of EZH2

Various inhibitors have been developed to achieve the competitive inhibition of the SAM binding to the SET domain of EZH2 [188]. These include EPZ-005687, EPZ-6438 (also named E-7438), developed by the pharmaceutics Epizyme [176]. Although both compounds are similar in their mechanism, EPZ-6438 has better properties regarding oral bioavailability in animals [176]. They preferentially inhibit the activity of EZH2 mutants (residues Y646, A682, and A692) found in B cell non-Hodgkin lymphoma [176]. In fact, EPZ-6438 is presently under study in a Phase I/II trial in solid tumors or B-cell lymphoma (Clintrial.gov identifier: NCT01897571) [176]. Recently Campbell *et al.* (2015) have published other molecule, EPZ-011989, whose difference with EPZ-6438 is a replacement of the second benzene ring and pyridine residue to increase the range of EZH2 inhibition, improving the pharmacokinetics and pharmacodynamics qualities, at least in a mouse xenograft model of human B cell lymphoma [177].

Other SAM-competitive inhibitors are EI1 from Novartis [178], and GSK126 from GlaxoSmithKline [179]. These two inhibitors caused loss of global genome H3K27 methylation, and reactivation of PRC2-silenced genes. Both act at low concentrations as antitumoral agents in diffuse large B-cell lymphoma cells (DLBCL), particularly when bearing EZH2 activating mutations. GSK126 has also been tested in mouse DLBCL xenograft [179]. GSK126 is currently under study Phase I clinical trial in adult patients of follicular lymphoma and relapsed diffuse large B cell lymphona (Clintrial.gov identifier: NCT02082977). Another derivative, GSK343 has shown preclinical antitumoral activity in three-dimensional culture of epithelial ovarian cancer [181].

A dual inhibitor of EZH2 and EZH1 (UNC1999) has also been developed [183]. It is structurally equivalent of GSK343, and is effective in DLBCL cells. Importantly, its EZH2/1 dual activity has a potential benefit in Mixed Lineage Leukemia (MLL) cancer cells [182].

Constellation Pharmaceuticals have developed various compounds with potential EZH2 inhibitory activity. After a general screening in search for potential inhibitors inducing genome-wide histone H3K27me3 reduction with activity at nanomolar concentration, they identified the lead compound CPI360 [196]. CPI-360 also acts through SAM-competition, reducing global H3K27me3 and H3K27me2 levels depending on the dose, but not affecting the protein levels of PRC2 subunits or the global levels of other tri- and dimethylation marks [184]. It has demonstrated activity against Y641N mutant EZH2-containing Germinal Center B Cell-like Diffuse Large B Cell Lymphoma cells (GCB-DLBCL) [184]. Unfortunately, complete target coverage could not be completed due to inadequate pharmacological properties [184]. Afterwards, CPI-169, a more potent EZH2 inhibitor, was identified with improved stability. CPI-169 showed effectiveness also in wt-EZH2 on GCB-DLBCL cells [197]. 

### 4.3. Other Approaches

The ability of SAH to inhibit the catalytic activity of EZH2 has been exploited by various groups trying to avoid the general effects attributable to overall SAH accumulation. One of them consists on the generation of stabilized peptides mimicking the α-helix of EZH2. This domain recognizes the corresponding domain of EED, therefore promoting the dissociation of the EZH2/EED complex, and impairing the function of PRC2 [185]. Detailed studies in comparison with GSK126 demonstrated a potential different mechanism, which caused growth arrest and induced differentiation of MLL-AF9 leukemia cells [185]. Nonetheless, a major caveat of this approach is the utility of these stabilized peptides *in vivo*. Indeed, while they are easily internalized by the cells *in vitro*, an *in vivo* investigation is still needed. In addition, as mentioned above, some EZH2 functions are independent on the binding to other PRC2 components. Consistently, these peptidomimetics approach will not impair these activities.

A series of compounds, also based on the structure of SAH, have been recently developed by Pfizer researchers [198]. Importantly, some of these newly characterized compounds displayed improved selectivity against EZH2 compared with the inhibition produced by SAH, without significant effects on other methyltransferases [198]. 

As mentioned above, EZH2 is also regulated by proteasome-mediated proteolysis [57,75,78,79,199]. Consequently, a possible approach to inhibit PRC2 activity is to increase this EZH2 degradation. Emodin (6-methyl-1,3,8-trihydroxyanthraquinone) is a natural product derivative that possesses multiple anti-cancer effects [200], and downregulates EZH2-mediated H3K27 trimethylation in BC cells [201]. Subsequently, novel Emodin derivatives have been obtained. Among them, NSC745885 have shown to cause EZH2 downregulation by increased proteasome-mediated degradation in BC cells, but not in immortalized non-tumorigenic bladder cells, and to suppress tumor development *in vivo* [186]. Nonetheless, considering the wide use of proteasome inhibitors as antitumoral agents, the observed finding of NSC745885 mediating increased proteasome degradation of EZH2, would require extensive studies to avoid possible side off effects.

Finally, another potential approach is to induce the expression of miRNAs targeting EZH2. Such approach has been reported for the natural product derivatives Gambogic Acid (GA) and Methyl Jasmonate (MJ). They are both considered promising therapeutic agents against various cancer types including prostate, pancreas, gastric, breast, and lung cancer, and their combination causes synergistic inhibitory activity. Interestingly, in a recent detailed study focused on BC cells, it has been shown that MJ significantly sensitized BC cells to GA-induced growth inhibition and apoptosis [187]. The plausible mechanism of this effect was attributed to EZH2 downregulation mediated by the increased expression of miR-101. Although a detailed molecular basis of such increased expression of miR-101 is still missing, the GA plus MJ combination exerted remarkable antitumor inhibitory activities in xenograft mouse models of human BC [187], thus demanding possible extensive studies on this combination for the possible future management of BC.

The role of EZH2 in chemotherapy resistance is to be determined in BC, although recent studies showed that acquired cisplatin resistance in ovarian cancer appeared linked to overexpression of EZH2 [202,203], in clear cell renal cell carcinoma models [204] or in lung adenocarcinoma [205]. In addition, increased EZH2 expression has been demonstrated to appear associated with worse outcome to tamoxifen therapy in metastatic breast cancer [206]. Intervention of EZH2 also inhibited the growth of Temozolomide-resistant glioblastoma cells [207].

## 5. Concluding Remarks

EZH2 plays a main role on the maintenance of cellular epigenetic integrity, being highly relevant on human BC, among other tumors such as prostate, breast, ovarian, renal carcinoma, lung, liver, brain, gastric, esophageal, pancreatic, or melanoma. As a main player within cancer epigenetics, it can become a promising tool for detection, diagnosis, prognosis, and prediction of response to possible therapies. Nevertheless, and in spite of the wide knowledge on EZH2 in normal development and in pathological conditions, there are still a large number of unanswered questions, which might be particularly relevant in BC and other cancers, where EZH2 upregulation appears to dictate the malignant progression. For instance, it is mandatory the identification of EZH2 target genes that are directly responsible for such oncogenic progression. In addition, it is interesting to determine whether EZH2 functions independent on histone methyltransferase are of relevance in these tumors. Similarly, whether the inactivation of KDM6A is equivalent to EZH2 overexpression is still unknown. There is still paucity in studies of EZH2 inhibitors in solid tumors, including BC. In these studies, the reported differences between long term EZH2 depletion as antitumoral approach *in vivo* using genetic technologies [208] or short-term EZH2 inhibition, such as that obtained with pharmacological approaches, should be deeply considered, to avoid reactivation and progression of the tumors [208]. Another aspect still obscure is the possible use of other inhibitors that may account of a partial EZH2 inhibition. As mentioned, the interaction of EZH2 with DNMTs or EHMT may account for some of the oncological properties of EZH2, and whether the inhibition of these partners also results in reduction of overall EZH2 activity is still unknown.

In summary, although our knowledge on the molecular mechanisms of EZH2 in relation with malignant transformation has been greatly improved in the recent years, there is still a lot of missing information necessary for full establishment of clinical settings of using this molecule as a target for cancer patients, in particular in BC.
